# Supplementary material for: Are carbon emissions trading and green financial instruments synergistic? -Comprehensive quantitative research based on content analysis
Source: PLoS One. 2024 Mar 7;19(3):e0298601. doi: 10.1371/journal.pone.0298601 (PMC10919601; doi:10.1371/journal.pone.0298601)
Supplement: S1 Data — (ZIP) [file pone.0298601.s001.zip › Data/地级市Z_F工作报告.zip!/║╙─╧/╓ú╓▌╩╨/└┤╫╘╣1⁄2╓┌║┼í╛┬φ┐╦╩2╛▌═°í┐_2012.docx]

各位代表：

现在，我代表市人民政府，向大会作政府工作报告，请予审议，并请各位政协委员提出意见。

一、2011年工作回顾

2011年是我市全面实施“十二五”规划的开局之年，是郑州都市区建设的起步之年。一年来，在省委、省政府和市委的正确领导下，我们紧紧抓住中原经济区上升为国家战略的重大机遇，谋划并实施了郑州都市区发展战略，明确了中原经济区核心增长区、全国重要的区域性中心城市和最佳宜居环境城市的战略定位，绘就了郑州未来发展的宏伟蓝图，激发了广大人民群众干事创业的热情，开启了郑州发展的新篇章。全市上下深入贯彻落实科学发展观，坚持“四个重在”实践要领，以郑州都市区建设为载体，按照“提速度、破瓶颈、抓统筹、上档次”的要求，团结拼搏，克难攻坚，顺利完成了市十三届人大三次会议确定的各项目标任务，实现了“十二五”发展的良好开局。初步统计，全市实现生产总值4912.7亿元，增长13.2%，分别高于全省、全国1.5个和4个百分点。全口径财政收入达到1305亿元，增长32.9%；地方财政总收入完成820亿元，增长27.6%；地方财政一般预算收入达到502亿元，增长30%。规模以上工业增加值完成2340亿元，增长22%。全社会固定资产投资完成3002.5亿元，增长24.3%。社会消费品零售总额完成1987亿元，增长18.4%。城镇居民人均可支配收入达到21612元，增长14.4%；农民人均纯收入达到11050元，增长19.8%。

郑州都市区建设开局良好。郑州都市区空间发展战略规划中期成果通过专家评审，土地开发利用、综合交通体系、产业布局等各类专项规划加快编制。制定实施了郑州都市区建设行动计划，郑州新区建设成效显著，中心城区功能得到提升；航空城、新郑新城、中牟新城、巩义新城、新密（曲梁）新城、登封新城融城发展进展顺利；宜居教育城、宜居健康城、宜居职教城等城市组团总体规划方案初步确定，基础设施和部分项目建设已经启动。坚持交通道路建设先行，积极构建郑州都市区路网骨架，中心城区连接“六城十组团”快速通道建设加快实施，完成出入市口征地拆迁110万平方米，绿化251万平方米。“六城十组团”建设完成固定资产投资950亿元。

产业结构调整成效明显。战略性支撑产业持续壮大。汽车及装备制造业快速发展，东风日产郑州60万台发动机工厂、宇通商务车和校车等项目加快推进，全年汽车整车产量达到35.5万辆，郑煤机高端产业园、中铁盾构、新大方重工等项目建设提升了装备制造业的层次和水平。物流商贸业快速发展，新加坡国际物流产业园、郑州新郑综合保税区物流园等物流基础设施项目建设步伐加快，中原万达广场等城市综合体丰富了商贸业态。电子信息产业不断壮大，富士康电子、格力电器等投资规模大、科技含量高的龙头企业带动作用凸显。文化创意旅游业发展迅速，国家动漫产业发展基地（河南基地）、郑州信息创意产业园等项目建设进展顺利，全市实现旅游总收入590亿元，增长15.7%。战略性新兴产业初具规模。新一代信息技术、生物医药、节能环保和新能源客车等产业加快发展，战略性新兴产业产值突破1000亿元，成为新的经济增长点。传统优势产业持续提升。铝及铝加工、纺织服装、食品、建材耐材等产业改造步伐加快，产业技术和产品档次显著提高。金融、会展、房地产业稳步发展。四方达、好想你、明泰铝业等6家企业顺利上市，上市企业达到35家。成功引进东亚银行，郑东新区金融集聚核心功能区建设加快推进。举办大型展会141场，展览面积达到155.3万平方米。房地产业在调控中健康发展，全市商品房实现销售额887.9亿元，增长14.9%。产业集聚区加快发展。全市产业集聚区营业收入完成 5150亿元，增长40%；固定资产投资完成1033.5亿元，增长62%。

改革开放迈出新步伐。各项改革进一步深化。国有企业改制工作稳步推进，战略重组工作取得实质性进展。郑州银行、百瑞信托实现增资扩股。行政审批制度改革加快，企业投资项目备案权限下放到所有县（市、区）和省级产业集聚区。政府机构改革顺利完成，事业单位分类改革有序展开，文化、教育、科技等领域改革继续深化。认真落实鼓励、支持和引导非公有制经济发展的政策措施，民营经济规模不断壮大。招商引资和对外开放成果丰硕。郑州新郑综合保税区、河南保税物流中心和出口加工区等对外开放平台建设取得重大成效。全年实际利用外商直接投资31亿美元，增长63%；引进市外境内资金941.6亿元，增长30.5%。市属及以下进出口总额完成153.6亿美元，增长239.5%，其中出口94.1亿美元，增长182.9%。积极承接产业转移，全年新开工外来投资项目152个，投资总额1454亿元。

城市形象显著提升。文明城市创建结出硕果。全市动员，全民参与，经过10年坚持不懈的努力拼搏，市民文明素质和城市文明程度得到显著提升，我市跨入全国文明城市行列，充分展示了郑州新的城市形象。郑州新区品牌形象凸显。郑州新东站综合交通枢纽工程加快建设，郑州新郑综合保税区热源厂一期工程投入运营，龙湖引黄调蓄工程全面启动。郑州新区完成基础设施投资160亿元，成为展示河南和郑州形象的窗口，成为承接产业转移的平台，成为宜居宜业宜商的福地。“畅通郑州”工程扎实推进。轨道交通1号线一期顺利铺轨，2号线一期开工建设，京沙快速通道部分路段、郑民高速郑州段建成通车。出入市口互通立交、三环快速化工程、南水北调跨线桥梁等一批重大项目开工建设。新增公共停车泊位2.5万个，新购置公交车953辆，郑州绕城高速免费通行。深入开展占道经营、交通秩序等专项整治活动，城市管理取得明显成效。综合交通枢纽地位不断强化。郑焦、郑汴城际铁路加快建设，郑州黄河公铁两用桥主体、火车站西广场等重点工程基本完工。郑州机场旅客吞吐量突破1000万人次。城市承载能力不断增强。刘湾水厂开工建设，实施五龙口、马头岗污水处理厂升级改造，城市供热、供气、供水等公共管网进一步完善。加快推进“数字郑州”建设，城市基础地理信息数据库基本建成。生态环境明显改善，常西湖生态公园等项目顺利实施，人民广场建成开园，市区新建绿地622万平方米，完成造林23.7万亩，新建林业生态乡（镇）8个、林业生态村100个。扎实开展截污攻坚工作，实施东风渠上游、潮河上游等河道治理工程，河道水质明显改善。节能减排力度加大。全年单位生产总值能耗下降3.7%，二氧化硫排放量和化学需氧量分别控制在10.18万吨和10.62万吨以下。城市集中饮用水源地水质达标率为100%，城区空气质量优良天数达到318天。

农村经济稳步发展。农业综合生产水平显著提高。粮食生产连续9年喜获丰收，总产量达到166.7万吨。畜牧业综合生产能力持续提高。大力实施“菜篮子”工程，完成新菜田建设3.6万亩。农村生产生活条件持续改善。实施异地扶贫搬迁和整村推进扶贫，1.67万人实现脱贫。实施自来水村村通工程，解决了43.9万人农村饮水安全问题。改造中低产田20万亩，升级改造农田灌溉机井8700眼。新建、改造农村道路578公里，新增农村户用沼气1万座，新建镇、村地埋式垃圾中转站611座，实施厕所无害化改造8.6万户。

民生保障水平持续提升。加大财政投入，较好地完成了省、市民生实事，解决了一大批群众关心的热点、难点问题。就业工作取得明显成效。全年新增城镇就业再就业13.8万人，农村劳动力转移就业12.6万人。发放小额担保贷款15.5亿元，促进了就业和再就业。社会保障体系不断完善。覆盖城乡的基本养老制度基本建立，城乡居民社会养老保险参保人数达到247万人。城镇居民基本医疗保险参保人数达到262万人，新农合参合人数达到417.4万人，参合率达到98.3%。调高了最低工资标准。物价上涨势头得到初步遏制。保障性住房建设进展顺利。优先安排土地供应，着力保障资金需求，强力推进项目建设，开工保障性住房5.57万套。

各项社会事业全面推进。自主创新能力不断提升。新建各级各类研发中心225家，其中国家级7家、省级57家。高新技术产业产值达到2806.7亿元，增长33.9%；增加值达到859.8亿元，增长29.2%。专利授权量6300件，增长11%，科技进步对经济增长的贡献率达到56%。连续5次荣获“全国科技进步先进城市”称号，建立了首个国家级知识产权创意产业试点园区。优质教育、医疗资源促进计划扎实推进。新建改扩建中小学校33所、幼儿园119所，新增优质中小学招生学位2.1万个，为全市所有中小学校开通了现代远程教育“班班通”学习站点。新建改扩建优质医疗机构10家，新增床位2250个。文化、体育等事业发展取得新成效。郑州市图书馆新馆、郑州商都遗址公园等项目建设顺利。新建农村文化大院200个、农家书屋750个。《水月洛神》荣获第八届中国舞蹈艺术最高奖“荷花奖”金奖第一名，《斗笠县令》获得中国戏剧节最高奖项。全民健身运动蓬勃发展，竞技体育水平不断提高，在第七届全国城市运动会上再创佳绩。人口与计划生育工作稳步推进，“双提高”工程深入开展，低生育水平持续稳定，荣获“全国人口和计划生育先进单位”称号。妇女儿童事业、老龄事业和残疾人事业取得新进展。

民主法制和社会管理不断加强。主动接受人大及其常委会监督，支持政协履行职能，加强与各民主党派、工商联和无党派代表人士的联系沟通。办理人大代表建议581件，政协委员提案658件，办结率均为100%，满意率均达到98.5%。积极推进依法治市工作，制定政府规章9件，提请市人大常委会审议地方性法规草案3件。全面实施“六五”法制宣传教育和依法治理工作规划。认真落实党风廉政建设责任制，廉政建设取得新成效。积极推行网络行政，创新了政府管理模式。完善社会管理体制，整合社会管理资源，认真开展“社区建设年”活动，有效解决影响社会和谐稳定的突出问题。建立健全食品安全监管机制，集中开展食品、药品安全专项整治。深入推进“两基三体系”建设，强化责任落实，安全生产形势总体平稳。加强和创新社会管理，深化“四严一创”、“两打一清”等专项行动，深化社会治安综合治理，扎实推进平安郑州建设，社会大局和谐稳定。

国防教育和国防后备力量建设取得新成效。大力支持军队建设，认真落实军队转业干部安置、征兵工作等政策措施，积极推进军民融合式发展，军政军民团结进一步巩固。顺利完成第六次人口普查。统计、审计、侨务、对台事务、民族宗教、社会科学、地方志、档案、气象和地震监测、人民防空等工作取得新成绩。对口支援新疆哈密工作进展顺利。

一年来，我们集中力量谋划了一批事关长远发展的大事。郑州都市区建设整体启动，新型城镇化建设全面铺开，环城苗木花卉产业发展规划、森林公园体系总体规划、郑州汽车城总体规划等重大发展规划编制完成并通过专家评审。集中力量推进了一批增强发展后劲的要事。富士康、格力电器、东风日产等一批重大产业项目强力推进，轨道交通1号线、京沙快速通道、三环快速化等“畅通郑州”工程取得重大进展，常西湖生态公园、滨黄河森林公园等一批生态项目扎实推进。集中力量解决了一批事关当前发展的急事。城市管理综合整治活动深入开展，城乡面貌取得明显改观；煤炭企业兼并重组基本完成，产业集中度进一步提升；南水北调工程郑州段全线开工建设，丹江口库区移民安置“四年任务、两年完成”。集中力量办成了一批事关城市竞争力的盛事。文明城市创建“十年梦圆”，喜获“全国文明城市”荣誉称号；郑州新郑综合保税区正式运营，成为我国中部地区首个封关运行的综合保税区；郑州机场二期扩建工程获国家批准，在建设国内大型航空枢纽机场方面取得了重大突破。

各位代表！

在复杂严峻的形势下能取得这些成绩，确属不易。这是我市全面贯彻落实科学发展观的结果，是省委、省政府和市委正确领导的结果，是市人大、市政协监督支持的结果，是全市人民群策群力、务实重做的结果。在此，我代表市人民政府，向辛勤工作在各条战线上的广大工人、农民、知识分子、干部、人民解放军指战员、武警官兵、公安民警以及社会各界人士表示崇高的敬意和衷心的感谢！

回顾过去一年的工作，我们也清醒地认识到经济社会发展中存在的困难和问题：经济结构性矛盾还比较突出，城乡发展还不够协调，农业基础薄弱，土地、资金和环境资源制约依然突出；城市承载能力还需要进一步提升，市政基础设施建设滞后，城区交通等问题仍然比较突出；服务业发展缓慢，在三次产业中所占的比重下降；社会管理工作难度加大，社会稳定、安全生产等形势还依然严峻；政府自身建设有待进一步加强，行政效率还需进一步提高。我们将正视问题，着力从基层基础做起，从体制机制入手，认真研究解决。

二、2012年工作总体要求和主要目标

今年是实施“十二五”规划承上启下的重要一年，是全面贯彻省市党代会精神、推进郑州都市区建设的关键之年，做好全年工作意义重大。从总体上看，我们既面临着极为复杂的国内外经济形势挑战，也面临着重大机遇。对我们来说，最大的机遇在于中原经济区上升为国家战略，国家和省支持我市在重点领域和关键环节的改革上先行先试，郑州都市区建设将形成新的投资热点；最大的挑战在于世界经济持续低迷，国内经济下行压力加大，周边省会城市围绕市场、资金、资源、人才等竞争将更加激烈。因此，我们既要正视困难和挑战，始终保持清醒忧患，更要看到机遇和优势，增强必胜信心，以邓小平理论和“三个代表”重要思想为指导，深入贯彻落实科学发展观，全面贯彻党的十七大和十七届三中、四中、五中、六中全会及中央、省委、市委经济工作会议精神，推动经济社会发展取得新的更大的成绩。

今年政府工作的总体要求：以科学发展为主题，以加快转变经济发展方式为主线，以新型城镇化引领“三化”协调科学发展为路径，以中原经济区郑州都市区建设为载体，以富民强市为中心任务，坚持“四个重在”实践要领，抢抓机遇，持续求进，突出提升，务实发展，创新惠民，积极作为，以优异成绩迎接党的十八大胜利召开。

今年经济社会发展的主要预期目标：生产总值增长13%左右，公共财政预算收入增长15%，规模以上工业增加值增长17%，固定资产投资增长25%，社会消费品零售总额增长17%，市属及以下进出口总额增长50%，实际利用外商直接投资增长15%，居民消费价格涨幅4%左右，城镇居民人均可支配收入和农民人均纯收入均增长10.5%，人口自然增长率控制在6.5‰以内，节能减排完成省定目标任务。

实现今年的要求和目标，必须抢抓机遇，突出重点，牢牢把握经济发展的主动权，充分发挥龙头、重心和示范带动作用，努力在中原经济区建设中“挑大梁、走前头”。紧紧围绕持续求进，破瓶颈、增投资。坚持解放思想，与时俱进，开拓进取，继续保持经济社会发展好的趋势、好的态势和好的气势。统筹协调各方力量，在依法依规的前提下，着力破解土地、资金等要素保障制约。把项目投资作为总抓手，按照“持续求进”的要求，逆势提速，突出抓好组团、项目和投资，围绕组团上项目，围绕项目增投资，增加投资促发展，增强发展的后劲和潜力。紧紧围绕突出提升，重运作、提效率。不断探索发展规律，持续创新发展理念，加快转变发展方式，在转变中提升，在创新中提升，在开放中提升，主动在中原经济区建设中先行先试，敢于打破常规，着力在提高运作能力上下工夫，在运作中找机遇、求发展、提效率，努力走出一条以新型城镇化为引领的“三化”协调科学发展之路。紧紧围绕务实发展，转作风、优环境。树立务实重做的形象，把务实作为一种追求、作为一种品格、作为一种责任，转变作风，善抓落实，努力建设“最佳投资环境、最佳就业创业环境、最佳人居环境、最佳旅游环境” 城市，创新工作机制，不断优化发展环境，真正使郑州成为宜居之城、宜业之城、宜商之城和宜游之城。紧紧围绕创新惠民，办实事、促和谐。始终坚持以人为本，着力解决人民群众最直接最关心最现实的利益问题，逐步提高社会保障能力和水平，逐步提高城乡居民收入水平，把推动经济发展、保障和改善民生、促进社会和谐更好地结合起来，让人民群众更多地享受改革发展成果。

今年，我们将围绕中原经济区郑州都市区建设，重点抓好以下工作。

（一）充分发挥新型城镇化引领作用，提升郑州都市区建设发展水平。建设郑州都市区必须坚持组团发展的理念，按照“城乡统筹、城乡一体、产城互动、节约集约、生态宜居、和谐发展”的要求，以新型城镇化为引领，以交通道路建设、生态廊道建设、新型社区建设、城市组团起步区建设、中心城区功能提升和产业集聚区建设等六项工作为切入点，统筹推进“两核六城十组团”规划建设，构建郑州都市区空间发展格局。坚持规划先行，加快编制郑州都市区总体规划、产业规划和土地利用规划，统筹推进各专项规划、控制性详规和重点地区城市设计，实现都市区规划全覆盖。突出中心城区和郑州新区核心带动作用，按照各组团的发展方向、产业定位，加快推进综合交通、现代产业、生态环境、基础设施等支撑体系建设。以复合城市建设理念，加快推进“六城”向中心城区融城发展，按照中心城区标准规划建设航空城、新郑新城、中牟新城、巩义新城、新密（曲梁）新城、登封新城，依托产业集聚区建设，充分发挥区位和资源优势，加快建设主导功能突出的现代化宜居新城区。加快“十组团”建设，优先布局生态性、公益性项目，带动环境提升和人气提升。加快中心城区连接“六城十组团”的快速通道建设，打造生态廊道和水系景观。依托新城和组团建设，大力发展县域经济，为县域经济发展注入新的活力。按照政府主导的拆迁安置模式，有序开展城中村改造和旧城改造。坚持试点先行、逐步推开的原则，实施城郊村合村并城。坚持“分类指导、科学规划、群众自愿、就业为本、量力而行、尽力而为”的原则，积极稳妥地推进新型农村社区建设，实行集中连片开发，为组团和产业集聚区发展拓展更大空间。

（二）强化新型工业化主导地位，提升郑州都市区产业支撑能力。按照“科技含量高、信息化涵盖广、经济效益好、资源消耗低、环境污染少、人力资源优势得到充分发挥”的标准，牢牢把握实体经济这一基础，坚持做大总量和优化结构并重，强化新型工业化主导地位，为郑州都市区建设提供产业支撑。加快汽车、装备制造和电子信息等战略性支撑产业发展。实施汽车城规划，加快东风日产、宇通客车、郑州海马等企业扩能升级项目建设，力争全市汽车产量达到50万辆，销售收入达到800亿元；加快装备制造业基地建设，实现恒天重工新纺机等项目竣工投产，装备制造业销售收入突破1800亿元；实施电子电器城规划，推进富士康郑州基地、格力郑州产业园等重点项目建设，电子信息产业销售收入突破1000亿元。加快战略性新兴产业发展。培育壮大新材料、新能源、生物医药、节能环保、物联网等产业，巩固新材料产业优势，推进新能源汽车产业化，推动郑州国家生物产业基地建设，引导光伏产业有序发展，加快建设“国家传感网创新示范区”，力争在重大关键性技术上实现新突破。加快传统优势产业改造提升。建设全国铝精深加工基地、现代食品工业基地、纺织服装基地，力争建材耐材产业销售收入突破2000亿元、煤电能源产业销售收入达到1200亿元。开展国家级“两化”融合试验区建设，推进数字城市建设，支持中兴通讯产业基地、微软教育云计算产业园等项目建设。加快产业集聚发展。按照“四集一转”的要求，调整产业集聚区布局，加快产业集聚区整合提升。强化主导产业培育，综合运用规划、土地利用、项目审批、财税调节等手段，推动同类和关联项目集中布局。推动对外连接通道、环保、供电、生活等基础设施建设，提升集聚区综合服务能力。积极争取省专项资金支持，加快省级产业集聚区投融资平台建设。力争销售收入超200亿元的产业集聚区达到10个，其中超500亿元达到3个。依托产业集聚区，积极培育装备制造、电子信息、建材耐材、煤电能源4个千亿级产业；力争年销售收入超100亿元企业达到10个，其中超300亿元企业4个、超500亿元企业1个。加快民营经济和中小企业发展。制定进一步促进民营经济和中小企业发展的具体措施，发展壮大民营经济，支持小微企业健康发展。激活民间投资，鼓励和引导民营企业参与国有企业重组。完善民营经济和中小企业项目对接、人才培训等服务体系。采取有效措施，引导担保机构规范发展。积极推进节能减排。抓好钢铁、化工、建材、电力、铝等行业节能降耗，加大淘汰落后产能力度，提高企业资源综合利用水平。积极推行清洁生产，推进污水处理、电力行业脱硫脱硝等减排项目建设，削减污染物存量。

（三）加快推进现代服务业，提升郑州都市区发展活力。坚持服务业优先理念，在薄弱环节上求突破，在发展方式上求创新，在规模总量上求扩张，积极培育现代服务业，提升郑州都市区产业发展层次。不断优化服务业发展布局。加大政策扶持力度，用足用活服务业发展引导资金，支持服务业关键领域、薄弱环节发展和品牌建设，提升综合服务功能。规划建设物流、金融、文化、旅游产业集聚区，加快形成一批主导产业突出、各具特色、功能互补的现代服务业集聚区。支持中心城区高端服务业发展，不断壮大以总部经济、楼宇经济为主的城区经济规模。加快推进国际物流中心建设。充分发挥铁路港、公路港、航空港、物流港资源优势，支持新加坡国际物流产业园和华南城物流园等重大物流项目建设，优化物流功能区布局。大力促进商贸业发展。以开拓市场、丰富业态、扩大消费为主线，促进商贸业发展。引进和建设一批大型城市商业综合体，加快大型商品交易市场、批发市场向三环以外搬迁，改造提升一批特色商业街区。积极推进“万村千乡”市场工程，加快蔬菜流通体系建设，扩大社区便利店覆盖范围。加快区域性金融中心建设。推进郑东新区金融集聚核心功能区建设，打造金融总部、财富管理机构、金融后台服务和金融服务外包集聚区。加强与各类金融机构的战略合作，吸引金融机构后台服务中心入驻。大力发展证券、保险、基金等业务，支持地方金融机构跨区域发展。加快会展和旅游业发展。发挥会展资源优势，开发高档次的精品展和特色展，巩固提升郑交会、汽车后市场博览会等自办品牌展会，筹办好第十八届郑州全国商品交易会、第四届中国郑州农业博览会。提高旅游产业发展质量和水平，以嵩山少林、黄帝文化、沿黄生态为重点，推进旅游资源和旅游产品开发，建设一批国际国内知名的旅游集聚区。促进房地产业健康发展。落实国家房地产调控政策，增加普通商品房供给，鼓励“六城十组团”开发建设大型居住社区，满足居民自住和改善性购房需求。

（四）大力发展都市型现代农业，提升郑州都市区发展基础地位。积极发展高产、优质、高效、生态、安全的都市型现代农业，夯实郑州都市区发展的基础。加快农业结构调整。重点扶持蔬菜、水果、花卉等作物设施化生产，推进现代农业示范区建设。抓好“菜篮子”工程，新建标准化菜田6万亩。积极促进现代畜牧业发展，重点推进标准化规模养殖，建立完善畜禽养殖环保体系。积极实施旅游富民工程，大力发展休闲观光农业，推动休闲农业示范点提档升级。推进大宗食品检测和肉类食品溯源体系建设，加快建设乡镇农产品质量安全监管公共服务机构，提高农产品质量水平。大力推进农业产业化经营，支持农业龙头企业发展。增强农业综合生产能力。加大农田水利基础设施建设力度，推进新一轮小型水库除险加固、中小河流治理、水土保持等项目建设。加大农业综合开发力度，改造中低产田20万亩，治理水土流失面积80平方公里，继续实施农用机井升级改造工程，新增有效灌溉面积3万亩。实施新型农机具和新技术的推广及示范工程，进一步提高农业机械化水平。推动农产品物流市场建设，建立农产品信息发布制度。加强农业气象服务，增强农村防灾减灾能力。认真落实强农惠农政策，财政支出重点向农业农村倾斜。改善农村生产生活条件。加强农村水、电、路等基础设施建设，推进城乡公共服务均等化。用上自来水的农村群众新增40万人，加快新一轮农村电网改造升级步伐，新修、改建农村道路400公里。结合新型农村社区建设，因地制宜配建小型污水集中处理设施，做到同步设计、同步施工、同步投入使用。实施农村环境连片整治和生态乡村创建活动，改善农村村容村貌。加快新农村现代流通服务网络工程建设，优化农村购物环境。对居住在深山区的2000户贫困人口进行异地扶贫搬迁。配合做好南水北调工程建设工作，加快配套工程建设步伐，认真落实移民后期扶持政策。加强农业科技创新。继续推进基层农业技术推广服务体系建设，大力实施沃土工程，发展节水灌溉。健全农作物病虫害预警体系，逐步实现农作物病虫害专业化统防统治。建立新品种新技术示范田，积极发展现代种业。加强农业科技成果转化、科技企业孵化等工作。

（五）加强城市建设和管理，提升郑州都市区综合承载能力。按照适度超前的原则，以现代基础设施建设为重点，不断构筑功能完备、协调配套、高效便捷、支撑有力的郑州都市区基础设施网络。加快推进郑州新区建设。重点推进龙湖、龙子湖、综合交通枢纽、绿博—白沙组团等重点区域开发建设。加强基础设施建设，开工建设项目143项，完成投资110亿元。支持高校园区和职教园区建设，引进更多技术性、经营性高端人才，建设人力资源高地，为郑州都市区发展提供人才支撑。不断完善中心城区服务功能。优化中心城区形态布局，实施城市重点片区的更新改造。加快新力热电厂等热源项目建设，新增供热面积100万平方米。加快城市新区、产业集聚区电网建设和老城区电网改造。推进刘湾等4个自来水厂建设。新建改造市区燃气管网60公里，新增居民用户8万户。加快推进马头岗污水处理厂二期工程等项目建设。切实加强城市道路等市政设施维护与更新。加强现代综合交通枢纽建设。抓好大型航空枢纽建设，开工建设郑州机场二期工程，积极引进基地航空公司，保持客货运稳定增长。启动上街机场改扩建工程的前期工作。配合推进干线铁路建设，力争石武客专郑州段上半年建成投用、郑徐客专开工建设，积极推进郑渝铁路、郑济铁路前期工作，争取完成郑开、郑焦城际铁路线下工程，加快郑州至机场城际铁路施工建设。配合做好连霍、京港澳高速升级改造工作。持续加快“畅通郑州”建设。坚持公交优先，推进“公交都市”建设，开辟公交线路10条以上，新购置公交车500辆。强力推进“两环十五放射”道路建设，加快城市出入市口互通立交、京沙快速通道、三环快速化等重点工程建设，加快轨道交通1号线一期、2号线一期建设，开工建设1号线二期和4号线部分工程，做好其他线路前期工作。优化城市路网结构，解决金水路、花园路等城市主干道的拥堵问题，优化连接中心城区、郑州新区的支线路网，推进道路微循环和路口渠化工作，新增停车泊位5万个，理顺停车场管理体制，实行差别化收费，加快城市智能交通指挥调度体系建设，提升城市交通畅通水平。改善城市生态环境。推进常西湖生态公园、滨黄河森林公园、南水北调带状公园建设，规划建设郑州第二植物园、第二动物园等大型公园绿地，市区新增绿地700万平方米，新植乔木50万株。启动国家森林城市创建工作，加强造林绿化工作，抓好生态廊道、苗木花卉产业带、森林公园体系和森林组团改造提升等工程建设，完成造林19万亩，创建6个林业生态乡（镇）和100个林业生态村。实施生态水系工程，打好生态水系截污攻坚战，打造滨水生态景观。加强空气质量和水环境治理，城区空气质量优良天数达到310天以上，城市集中饮用水源地水质达标率达到98%以上。开展PM2.5监测和分析研究治理工作。强化城市管理。按照权责一致、重心下移的原则，确立城市区在城市管理中的主体地位，理顺城市管理体制。健全精修精养、应急服务、有偿整改等城市管理机制，提升城市管理效能。持续推进城市管理综合整治活动。切实加强城管执法队伍建设，提高依法行政、文明执法水平。

（六）继续推进改革开放，提升郑州都市区建设原动力。深化改革开放，实施开放创新双驱动战略，加快形成全方位、多层次、宽领域的开放新格局和自主创新体系。持续深化改革。主动加强与国家和省有关部门的沟通衔接，争取政策支持，在重点领域和关键环节改革方面先行先试。深化投融资体制改革，加快政府融资平台整合步伐，重点培育2至3家大型投融资企业集团；鼓励大型企业和城投企业发行债券，支持信托融资，积极发展融资租赁、股权投资和风险投资；大力支持企业上市，培育一批高成长性上市后备企业。深化国企改革重组，加快建立现代企业制度。深化行政管理体制改革，开展城市组团、产业集聚区和经济发达镇管理体制改革试点。深化农村改革，推动集体建设用地有序流转，引导促进农村土地承包经营权流转，开展农村集体林权、农村房屋抵押贷款试点，推进农业政策性保险工作。深化文化、教育、医疗卫生、财税等重点领域的改革。大力发展开放型经济。充分发挥综合保税区、保税物流中心、出口加工区 “三大平台”和航空、铁路、公路“三大口岸”的作用，高标准规划建设郑州国际陆港，打造内陆开放高地。积极承接国内外产业转移，深度参与国内外产业链分工调整。持续实施“走出去”战略，鼓励和引导优势行业开展对外合作，支持优势企业承揽大型境外工程项目并带动设备和技术出口，提高对外经济技术合作的质量和水平。加快引进一批国内外知名的服务外包企业，培育一批具有自主知识产权和自主品牌的本地服务外包骨干企业，积极申报“中国服务外包示范城市”。持续开展大招商活动。把招商引资作为开放工作的重中之重，围绕重大产业项目、企业总部、高端人才、研发检测平台等方面，按照产业发展规划，策划开展一系列招商活动，引进一批重大项目。重点做好280个在建、签约、在谈项目的推进落实工作，确保项目早建设、早投产、早见效，提升招商引资工作实效。加强自主创新体系建设。以建设创新型试点城市为载体，实施重大科技专项和科技工程。加大财政科技投入力度，加强研发平台建设，与国内外知名大学或科研院所建立战略合作关系，支持国家级重点实验室建设，促进产学研合作。加强科技创新服务体系建设，完善科技成果转化公共服务平台，重视创新型科技人才的培养。力争全市专利授权量突破8000件，高新技术产业产值达到3300亿元，科技进步贡献率达到57%。

（七）切实加强文化建设，提升郑州都市区软实力。发挥古都优势，加快建设华夏历史文明传承创新核心区，提升城市文化软实力。进一步提升全国文明城市建设水平。建立健全长效机制，巩固文明城市创建成果。扎实开展“道德讲堂”和志愿者活动，持续提升市民文明素质。实施城市公共文明指数测评，不断创新内容、创新载体、创新机制，推动郑州向更高水平的文明城市迈进。大力繁荣文化事业。完成国家公共文化服务体系示范区创建任务，深入推进文化惠民工程，各项指标达到或超过国家要求的中部地区标准。加快各级各类文化基础设施建设，建成郑州市图书馆新馆，推进郑州群艺馆东区新馆等重点项目建设。实施文化精品创作工程，推出一批在全国有较强影响力的精品力作。提升公共文化服务水平，支持各类企事业单位参与公共文化服务。加快发展文化产业。实施项目、科技和品牌带动，扶持影视动漫、演艺娱乐、出版发行等文化产业。规划建设一批文化产业园区、特色文化街区，加快郑州华强文化科技产业基地、国家动漫产业发展基地（河南基地）、郑州信息创意产业园等文化产业集聚区建设。扶持小樱桃、小小说等优势品牌加快发展。继续举办好黄帝故里拜祖大典、郑州国际少林武术节等活动。推进优秀历史文化传承创新。编制《大遗址郑州片区建设总体规划》，加快建设郑州大河村遗址、郑韩故城、西山古城等一批大遗址公园，加快建设郑州商都遗址博物院、郑州博物馆新馆等一批博物馆。加大登封“天地之中”历史建筑群保护管理和宣传推广。

（八）着力民生事业发展，提升郑州都市区保障能力。保障和改善民生，是政府工作的出发点和落脚点，也是打造郑州都市区和谐环境的重要抓手。强力推进民生工程。围绕与百姓生活息息相关的“柴米油盐酱醋茶、衣食住行医教保”，加大投入，办好省、市民生实事。千方百计扩大就业。发放小额担保贷款16亿元，促进以创业带动就业。新增农村劳动力转移就业10万人、城镇就业再就业13万人，城镇“零就业家庭”动态为零。大力开发劳动就业岗位，帮助高校毕业生、失业人员、进城务工人员等重点群体实现就业再就业。稳步增加居民收入。深化工资收入分配制度改革，促进形成合理有序的工资收入分配格局。落实好增加群众收入的政策措施，确保城乡居民人均收入两位数增长。完善机关事业单位工资和津补贴制度，加大奖励性绩效工资落实力度。落实最低工资标准，做好工资集体协商工作，促进职工收入与企业收入同步增长。认真落实国家和省政策规定，继续为企业退休人员增加基本养老金。着力加强社会保障体系建设。提高社会保险统筹层次，继续扩大社会保险覆盖面。深入开展城乡居民养老保险和职工工伤保险工作，做好被征地农民社会保障工作。完善基本养老金、城乡居民低保标准正常调整机制。推进残疾人保障体系和服务体系建设，保障困难群体的基本生活。加快保障性住房建设。新开工建设保障性住房5万套以上、竣工2.9万套。制定完善保障性住房分配、管理、退出等办法，确保公平分配。加快教育事业发展。实施优质教育资源促进计划，市区新增优质中小学招生学位2.36万个。市区新建、改扩建中小学校30所，六县（市）城区新建、改扩建中小学校10所以上。全市新建、改扩建幼儿园84所，扶持200所民办幼儿园达标升级，为符合条件的适龄幼儿发放学前教育补贴。深化学区制改革，推进多样化特色办学模式，促进义务教育均衡发展。加快市区职业教育资源整合重组，推动宜居职教城建设。大力发展民办教育，促进民办教育和公办教育协调发展。重视高等教育发展，提升郑州师范学院本科办学水平，力争郑州幼师升专工作取得实质性突破，加快郑州职业技术学院新校区等项目建设。不断提高人民群众健康水平。加快市三院北区医院、市儿童医院东区医院和宜居健康城项目建设进度。深化医疗卫生体制改革，实施优质医疗资源促进计划，建设区域性医疗中心。新农合人均筹资标准提高到290元，住院补偿封顶线由10万元提高到15万元。实施乡镇卫生院“温暖工程”。规范城市社区卫生服务机构和农村公共卫生管理服务中心建设，完善公共卫生服务体系。积极开展爱国卫生运动。大力发展妇女儿童事业、老龄事业和残疾人事业，制定实施新十年《郑州妇女发展规划》和《郑州儿童发展规划》。开展全民健身运动，不断增强群众身体素质。做好人口和计划生育工作，提高出生人口素质，促进人的全面发展。

（九）加强和创新社会管理，提升郑州都市区和谐水平。健全党委领导、政府负责、社会协同、公众参与的社会管理格局，在服务中实施管理，在管理中体现服务，全面提高社会管理科学化水平。健全源头预防的矛盾排查化解体系。完善社情民意调查分析机制，畅通群众诉求渠道，了解民情民意。落实社会稳定风险评估机制，完善“大调解”工作机制，认真解决好土地征用、城市建设拆迁、企业改制、涉法涉诉等领域的突出问题。规范担保公司运营，妥善化解金融风险。大力开展法律援助活动，扩大法律援助覆盖面，努力做到“应援尽援”。加大社会管理综合治理力度。健全社会管理组织机构，完善社会管理体制机制。推进平安郑州建设，依法打击黑恶势力、严重暴力和多发性侵财犯罪。加强社会管理治安防控体系建设，形成责任明确、程序规范、反应迅速的群防群治体系。进一步加大技防建设力度，努力实现技防全覆盖。完善应急管理体制，提高预知、预防、应急处置能力。支持新郑市开展全国社会管理创新综合试点工作。加强重点人群的管理服务。完善流动人口服务管理机制，逐步实现公共服务由户籍人口向常住人口扩展。着力解决流动人口在劳动就业、社会保障、子女入学、医疗卫生等方面存在的问题。鼓励支持公办养老机构对社会开放，引导社会力量参与养老事业，探索医养结合养老新模式，有效解决空巢老人独居问题。关注关爱留守流动儿童，新建 “留守流动儿童之家”40个。全面提升社区建设和服务水平。加强社区服务中心建设，推行县（市、区）直管社区体制试点，完善社区服务功能。深化“社区服务年”活动，统筹推进社区综合创建。拓展物业管理服务功能，实施老旧小区物业基础设施改造工程，健全物业公司信用评价、服务质量评价和末位淘汰机制，积极推进物业服务向新型农村社区延伸，逐步提升物业服务水平。完善社会安全体系建设。把安全放在发展的首位，推行安全生产“一岗双责”制，认真落实政府安全监管责任，强化企业主体责任，切实解决煤矿、非煤矿山、危险化学品、消防、道路交通等重点领域存在的突出问题。加强校车安全管理，全面落实校车安全责任制。加强综合协调能力建设，构建政府监管、行业自律、社会监督相统一的食品安全监管体系。开展规模以上餐饮服务企业信用达标升级活动。加强网络虚拟社会管理，持续净化网络环境。

继续做好国防动员、人民防空工作，加强全民国防教育和民兵预备役建设，深入开展双拥共建活动，促进军民融合式发展。巩固平等、团结、互助、和谐的社会主义民族关系，发挥宗教界人士和信教群众在促进经济社会发展中的积极作用。充分发挥工会、共青团、妇联等人民团体的桥梁纽带作用。扎实做好统计、审计、外事、侨务、对台事务、社科研究、地方志、档案和地震监测等工作。继续做好对口支援新疆哈密工作。

各位代表！

《中原经济区郑州都市区建设纲要（草案）》、《郑州市2012年民生“十大实事”（草案）》已印发会议，请予审议。

四、努力建设服务型政府

面对新的形势和任务，我们必须坚持用领导方式转变加快发展方式转变，更加注重政府自身建设，更加注重提高主动服务意识，更加注重提升政府公信力和执行力，积极作为，务实重干，奋发有为，努力建设服务型政府。

（一）转变职能，提高效率。围绕服务企业、服务群众、服务基层，加快政府职能转变，切实提高行政效率。推进政企、政事、政府与市场中介组织分开，履行经济调节和市场监管职能，为各类市场主体提供优质服务。强化社会管理和公共服务职能，维护社会公平正义。加大政务公开力度，积极推进财政预决算公开；加快建设政府信息公开网站，及时充实和更新信息发布内容，维护群众的知情权。充分发挥网络便捷高效优势，积极推进网络行政。深入推进“两转两提”，持续开展公务员培训和职业道德建设，积极开展争做“人民满意公务员”活动，努力建设一支政治坚定、业务精湛、作风过硬、人民满意的公务员队伍；切实提高行政决策效率、协调效率和执行效率，营造良好发展服务环境。深化行政审批制度改革，建立规范高效的审批运行机制，减少审批环节，压缩审批时间，优化审批流程，推动政府工作有序高效运转。

（二）依法行政，科学决策。严格依照法定权限和程序行使权力、履行职责，带头维护宪法和法律的权威。严格执行人大及其常委会的决议决定，依法接受其法律监督和工作监督，主动接受人民政协的民主监督，认真办理人大代表建议和政协委员提案，广泛听取各民主党派的意见。健全行政监督体系，强化责任追究制度，加强审计监督、行政监察和行政层级监督，严格行政问责。坚持开门立法，提高政府立法质量和水平。完善行政执法程序，规范行政执法行为。加强行政复议工作，强化行政执法责任和过错追究。建立健全政府决策机制，进一步提高依法决策、科学决策和民主决策水平。

（三）强化责任，务实重干。强化政府工作责任，全面推行岗位责任制、服务承诺制和限时办结制，狠抓各项工作落实，形成一级抓一级、层层抓落实的责任体系。完善重大事项和重点工作跟踪落实机制，确保政令畅通、令行禁止，提高政府执行力。坚持把务实重干贯穿于政府工作之中，大兴务实之风，落实“三具两基一抓手”，说实话、办实事、重实绩、求实效，责随职走、心随责走，力戒官僚主义、形式主义。改进工作方式方法，注重科学运作、有效运作。精简会议和文件，减少迎来送往，严格控制和规范各种检查、评比、达标活动。以务实重干求发展，以务实重干谋跨越，以务实重干求突破。

（四）勤政为民，廉洁从政。深入贯彻中纪委十七届七次全会精神，牢记“两个务必”，坚持以人为本、执政为民，不遗余力为百姓办实事。认真落实党风廉政建设目标责任制，严格执行廉洁从政各项规定，着力完善教育、制度、监督、问责并重的惩治和预防腐败体系，不断拓展从源头上预防腐败工作领域，强化对权力运行的制约和监督。加强政府公共资源管理，建设郑州公共资源交易中心。加大纠风和专项治理力度，切实解决损害群众利益的突出问题，确保人民赋予的权力真正为人民谋利益。严格控制行政经费等一般性支出，控制楼堂馆所建设。依法惩处各类违法违纪行为，营造风清气正、廉洁高效的政务环境。

各位代表！

郑州都市区建设的美好前景激励着我们，人民群众的热切期盼鼓舞着我们。让我们紧密团结在以胡锦涛同志为总书记的党中央周围，高举中国特色社会主义伟大旗帜，深入贯彻落实科学发展观，在省委、省政府和市委的正确领导下，抢抓机遇，开拓创新，紧紧依靠全市人民，以坚定不移的信念、勤政务实的作风、埋头苦干的精神，努力开创郑州都市区建设新局面，以优异的成绩迎接党的十八大胜利召开！
